# Severe Short Stature in an Adolescent Male with Prader-Willi Syndrome and Congenital Adrenal Hyperplasia: A Therapeutic Conundrum

**DOI:** 10.1155/2017/4271978

**Published:** 2017-05-30

**Authors:** Meredith Wasserman, Erin M. Mulvihill, Angela Ganan-Soto, Serife Uysal, Jose Bernardo Quintos

**Affiliations:** ^1^The Warren Alpert Medical School, Brown University, Providence, RI, USA; ^2^College of Human Ecology, Cornell University, Ithaca, NY, USA; ^3^Alexian Brothers Women and Children's Hospital and Amita Health Medical Group, Hoffman Estates, IL, USA; ^4^Division of Pediatric Endocrinology, Rhode Island Hospital and Hasbro Children's Hospital, The Warren Alpert Medical School, Brown University, Providence, RI, USA

## Abstract

Congenital adrenal hyperplasia (CAH) due to 21-hydroxylase deficiency results in excess androgen production which can lead to early epiphyseal fusion and short stature. Prader-Willi syndrome (PWS) is a genetic disorder resulting from a defect on chromosome 15 due to paternal deletion, maternal uniparental disomy, or imprinting defect. Ninety percent of patients with PWS have short stature. In this article we report a patient with simple-virilizing CAH and PWS who was overtreated with glucocorticoids for CAH and not supplemented with growth hormone for PWS, resulting in a significantly short adult height.

## 1. Introduction

Congenital adrenal hyperplasia (CAH) is a family of autosomal recessive disorders characterized by the inability to synthesize cortisol from cholesterol in the adrenal cortex. 21-hydroxylase deficiency is the most common cause of CAH and results in excess androgen production due to shunting of intermediates in the steroidogenic pathway toward androgen synthesis. Excess androgen production causes the early fusion of epiphyseal plates, which stunts growth. Treatment for patients with CAH involves the delicate balance of suppressing adrenal androgens while maintaining normal growth.

Short adult stature is common in 21-hydroxylase deficiency. Final adult height in patients with CAH is 1.38 SD lower than the population norm and their corrected height (final height minus genetic height potential) is 1.03 SD lower than their genetic height potential [1]. This short stature is seen in CAH patients even in the presence of early glucocorticoid treatment to suppress excess androgen production. Excess glucocorticoids can inhibit the pituitary production of growth hormone and contribute to short stature [2]. Growth hormone therapy was found to improve final height in patients with CAH by 9.2 cm ± 6.7 cm in males and 10.5 ± 3.7 cm in females [3]. It was also found that treatment with growth hormone can counter the growth-suppressing effects of glucocorticoids [4].

Prader-Willi syndrome (PWS) is a genetic disorder resulting from a defect within the Prader-Willi critical region on chromosome 15 due to paternal deletion, maternal uniparental disomy, or imprinting defect. PWS is characterized by short adult stature, morbid obesity, hypogonadism, and characteristic facial features such as narrow bifrontal diameter, almond-shaped eyes, and small mouth with downturned corners and thin upper lip [5]. Decreased growth hormone levels and serum levels of insulin-like growth factor 1 (IGF-1) and insulin-like growth factor binding protein 3 (IGFBPG-3) are found in 40–100% of children with PWS [6, 7]. Additionally, 90% of patients with PWS have short stature [7]. In the absence of growth hormone treatment and subsequent pubertal growth spurt, average adult height in male and female patients with PWS is 155 cm and 148 cm, respectively [7]. Patients treated with growth hormone were found to attain a mean adult height SDS of −0.3 ± 1.2 [8]. In addition, growth hormone treatment has been shown to improve body composition by lowering body fat percentage and increasing lean body mass [9].

To our knowledge, no case of simultaneous PWS and CAH has been reported. Here, we report a patient with PWS and CAH who was overtreated with glucocorticoids for CAH and not supplemented with growth hormone for PWS, resulting in a significantly short adult height.

## 2. Case Report

We report the case of a 20-year-old Puerto Rican male with PWS and CAH who we initially evaluated when he was 17-year-old. He was born full term with birth weight 5 pounds, birth length 21 inches, and physical exam showing bilateral cryptorchidism and hypotonia. Karyotype is 46XY. He experienced feeding difficulties in the first year of life and had delayed milestones. At 3 years of age, he developed pubic hair and his laboratory examination showed 17-hydroxyprogesterone level 8,676 ng/dL and DHEAS 29 ug/dL. He was diagnosed with CAH and prescribed hydrocortisone. He was treated with fludrocortisone for the first 1-2 years after diagnosis. At 9 years of age his dose of hydrocortisone was increased, most likely due to poor compliance to treatment and poor adrenal control. Over the years he struggled with obesity and learning and behavioral problems, including hyperactivity and short attention span. He was diagnosed with PWS at 12 years of age, showing a submicroscopic deletion of chromosome 15 (q11.2q11.2). He was not treated with growth hormone. He also had a bone age of left hand in an outside hospital when he was 12 years old and his bone age was reported as 16 years old.

**Figure 1:**
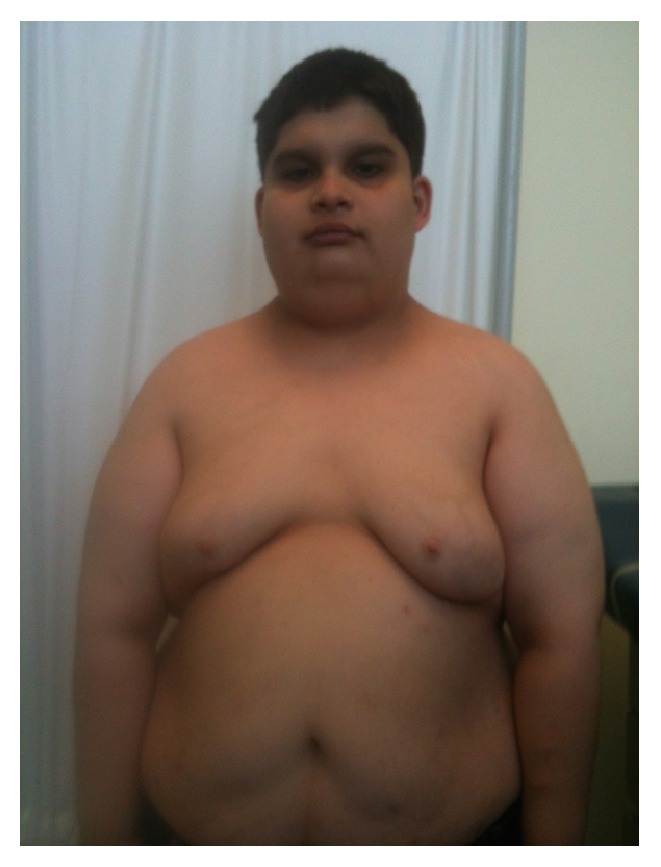
Characteristic facial features of Prader-Willi syndrome.

**Figure 2:**
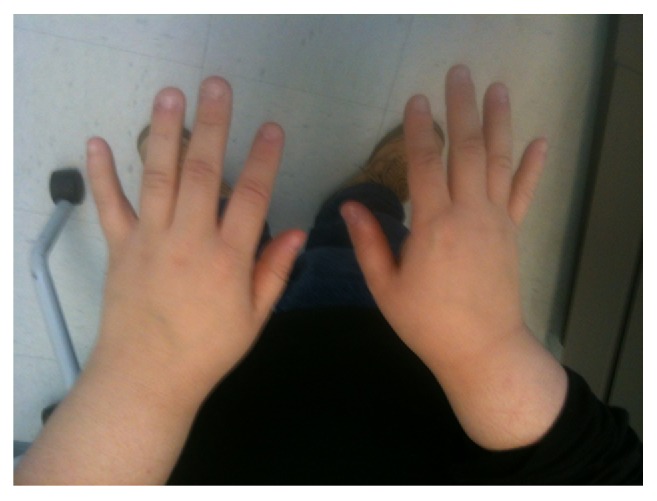
Small hands characteristic of Prader-Willi syndrome.

He came to our clinic for the first time at age 17 years. He was taking hydrocortisone 40 mg in the morning and 20 mg in the evening (equivalent of 37.5 mg/m^2^/day). Usual dose of hydrocortisone is 10–20 mg/m^2^/day. Physical exam showed a short male with almond-shaped eyes, fair skin, narrow bifrontal diameter, upslanted palpebral fissures (Figure 1), and small hands (Figure 2). His height was 138.6 cm (<5 percentile; −5.3 height SDS), height age 10 years, weight 72.3 kg (72nd percentile; 0.6 SDS), BMI 37.48 kg/m^2^ (>99th percentile), and blood pressure 124/56 mmHg. He was Tanner 3 for pubic hair and genitalia and testicular volume was 5-6 cc. His mother measured 159.8 cm and his father's reported height is 167.6 cm. Based on parental heights his midparental target height is 170.2 cm.

Laboratory investigation showed 17-hydroxyprogesterone 16 ng/dL, testosterone 5 ng/dL (normal for 17-year-old male is 348–1197 ng/dL), and renin 10.47 ng/mL/hr. Adrenal hormone assays were done by liquid chromatography tandem mass spectrometry at Esoterix Laboratory Services in Calabasas Hills, California, United States. Bone age of left hand was 17 years of age, consistent with his chronological age.

We decreased his hydrocortisone dosage to 20 mg twice a day (equivalent 25 mg/m^2^/day) with the goal of 17-hydroxyprogesterone level between 100 and 1000 ng/dL and androstenedione level normal for age and sex. His electrolytes were Na^+^ 135 mEq/L, Cl^−^ 100 mEq/L, CO_2_ 27 mEq/L, and K^+^ 4.3 mEq/L. Hologic DEXA scan showed bone mass density below expected range for age. Femoral neck *Z*-score was −3.2, total hip *Z*-score was −3.3, and total body *Z*-score was −1.7 (−1.0 to −2.5 SDS).

He returned to our clinic six months later and his laboratory work-up showed 17-hydroxyprogesterone 23 ng/dL, androstenedione < 10 ng/dL (normal 17–72 ng/dL for Tanner 3), renin 3.43 ng/mL/hr, IGFBP-3 2.8 mg/L (normal 2.5–4.8 mg/L), and IGF-1 252 ng/mL (normal 161–467 mg/mL, mean 290 mg/mL). We further decreased hydrocortisone dose to 20 mg in the morning and 10 mg in the evening (equivalent 18 mg/m^2^/day), which resulted in normal adrenal control (17-hydroxyprogesterone 426 ng/dL). Pituitary gonadal axis evaluation showed LH 9.6 mIU/mL, FSH 21 mIU/mL, testosterone 139 ng/dL, and androstenedione 35 ng/dL, suggesting primary gonadal dysgenesis. Patient has not had testicular ultrasound to evaluate for testicular adrenal rest tumor (TART). An ACTH stimulation test to confirm the CAH diagnosis was done after stopping hydrocortisone for 24 hours. Results were consistent with simple-virilizing CAH due to 21-hydroxylase deficiency (Table 1). Genetic testing for mutations in the 21-hydroxylase gene has not been obtained. Testosterone cypionate 50 mg IM every four weeks was started. This resulted in more aggressive behavior and violent outbursts prompting discontinuation of testosterone treatment.

## 3. Discussion

We report the case of a 20-year-old male with simple-virilizing CAH and PWS, who probably had both periods of undertreatment and overtreatment of glucocorticoids and lack of growth hormone treatment for PWS, resulting in a significantly short adult height. He also displayed aggressive behavior after being treated with testosterone for hypogonadism associated with PWS. To the best of our knowledge, there has been no other reported case of a patient with both PWS and CAH.

The chance of one patient having both CAH and PWS is extremely rare. The worldwide incidence of severe classic CAH is 1 in 15,000 [10] and the incidence of PWS is estimated to be between 1 in 10,000 and 1 in 30,000 [11].

The late diagnosis of PWS and missed opportunity for growth hormone therapy contributed to his significantly short adult stature. His height is 138.6 cm, or −5.3 height SDS. The average adult height of a male with untreated PWS is 155 cm (−3 height SDS) [7]. In one study, PWS patients treated with growth hormone achieved a mean adult height of −0.3 ± 1.2 SDS, while untreated patients achieved a mean adult height of −3.1 ± 1 SDS [8]. A retrospective study evaluating the effects of hydrocortisone treatment in patients with classical CAH showed that higher doses of glucocorticoids in children with CAH may result in decreased linear growth [12]. Additionally, a study of children with classic CAH found that the average final height for males treated with an average daily hydrocortisone dose of 19.7 ± 2.9 mg/m^2^/day was 163.1 ± 6.6 cm [13].

We hypothesize that periods of undertreatment and overtreatment with hydrocortisone contributed to his severe short stature. Factors that cause short adult stature in patients with 21-hydroxylase deficiency are (1) elevated adrenal androgens, which cause advanced epiphyseal maturation and premature epiphyseal fusion, (2) early or precocious puberty, which also leads to premature epiphyseal fusion, and (3) overtreatment with glucocorticoids [14]. Our patient also experienced premature epiphyseal fusion, as his bone age was 16 years at a chronological age 12. This premature epiphyseal fusion could be a result of periods of glucocorticoid undertreatment for CAH. Additionally, overtreatment with glucocorticoids most likely led to growth hormone suppression leading to poor growth velocity. This case reinforces the importance of growth hormone treatment for PWS and reduced glucocorticoid treatment for CAH, especially when both disorders coexist in one patient. Additionally, our patient was born small for gestational age with a birth weight of 5 pounds. It was unclear if he had catch-up growth due to lack of growth charts from the early childhood period. This may also have contributed to his short stature.

Our patient's elevated FSH and LH levels and low testosterone levels indicate that he has primary hypogonadism. Eiholzer et al. found that boys with PWS have a combination of central and primary hypogonadism involving deficiency of LH and testosterone secretion at puberty and damage of the seminiferous tubules and Sertoli cells, resulting in reduced inhibin B levels and elevated FSH levels [15]. Additionally primary hypogonadism may be due to TART. Vanzulli et al. reported a prevalence of 27% of TART in a group of 30 CAH patients with age range 9 to 32 years [16]. It is more commonly seen in patients with salt-wasting CAH; however TART also occurs in patients with simple-virilizing and nonclassical CAH.

He displayed aggressive and violent behavior after testosterone replacement therapy, prompting discontinuation of therapy. It has been suggested that, to avoid aggressive behavior, testosterone should be administered daily as gels or patches instead of monthly depot intramuscular (IM) injections [17, 18]. Depot testosterone preparations cause unpredictable peaks and troughs, which may cause mood instability. Others suggest reducing the testosterone dose for patients with PWS to one-third to one-half of the normally recommended dose [6].

This unique case highlights the importance of careful follow-up and monitoring of adrenal androgens in a patient with CAH. Glucocorticoid dosage should be adjusted to prevent premature epiphyseal fusion and to maximize growth. This case also highlights the importance of early diagnosis of PWS and initiation of growth hormone treatment. When hypotonia, feeding difficulties followed by obesity, characteristic facial features, and characteristics of hypogonadism present in an infant, PWS should be suspected [5]. This case also exemplifies the need for further research on appropriate testosterone therapy for males with PWS.

## Figures and Tables

**Table 1:** Results of high dose ACTH stimulation test with 250 mcg cosyntropin.

Time (mins)	Cortisol (ug/dl)	17-Hydroxyprogesterone (ng/dl)	Androstenedione (ng/dl)	Testosterone (ng/dl)	DHEA (ng/dl)
0	2.9	9,410	106	31	<20
60	2.7	11,000	77	44	<20
